# The role of N-glycosylation of CD200-CD200R1 interaction in classical microglial activation

**DOI:** 10.1186/s12950-018-0205-8

**Published:** 2018-12-19

**Authors:** Chao Liu, Yifen Shen, Ying Tang, Yongchun Gu

**Affiliations:** 1 Central Lab, First People’s Hospital of Wujiang Dist, Suzhou, 215200 Jiangsu Province China; 2 Jiangsu Key Laboratory of Oral Diseases, Nanjing Medical University, 136 Hanzhong Road, Nanjing, 210029 Jiangsu Province China

**Keywords:** CD200R1, Microglial activation, CD200, Lipopolysaccharide, Neuron

## Abstract

**Background:**

Microglial inflammatory activation is the common feature of the central nervous system (CNS) diseases. Microglia can be activated and particularly polarized toward a dual role in the injured CNS. The CD200 receptor 1 (CD200R1) inhibits inflammatory microglia activation as illustrated by studies. Publications show abnormal activation of microglia secondary to the deficient inhibit of CD200-CD200R interaction. In the present study, we established a neuronal-microglia co-culture system to investigate the association between CD200R1 engagement and classical microglial activation. We analyzed the glycosylation of CD200R1 and the CD200 binding. Secretion of pro-inflammatory cytokines were measured.

**Results:**

CD200R1 was N-glycosylated at Asparagine 44 (Asn44, N44). Mutation of this site disrupted CD200-CD200R1 interaction and up-regulated the expression of cytokines iNOS, CD86, IL-1β and TNF-α.

**Conclusion:**

N44 of CD200R1 is a significant binding site for CD200-CD200R1 interaction and play a critical role in the maintenance of microglia. The N-glycosylation of CD200R1 could serve as a therapeutic agent for CNS inflammation.

**Electronic supplementary material:**

The online version of this article (10.1186/s12950-018-0205-8) contains supplementary material, which is available to authorized users.

## Background

Inflammation is a shared characteristic pattern of degenerative central nervous system (CNS) disorders, infectious diseases and immune diseases [1]. Neuroinflammation executes dual detrimental and beneficial effects on the outcome of Janus-faced microglial activation [2, 3]. Microglial cells are involved in inflammatory and immune responses. They are resident macrophages of the CNS, acting as sensors of pathologic events, involved in the scavenging of cell debris and pathogens, and contributing to promote regrowth and remapping [4]. Microglial activation in the CNS is heterogeneous, which can be described as a functional dichotomy, termed classical (M1) and alternative (M2) activation. They play critical roles in re-establishing brain homeostasis and minimizing neuronal damage [2]. Classical activation is induced by Th1 cytokines lipopolysaccharide (LPS), interferon-γ (IFN-γ) or other bacterial products, whereas alternative activation is induced by Th2 cytokines IL-4, IL-13, or IL-10 [5, 6]. Microglia in classical activation state named “M1 microglia” induce increased synthesis and expression of pro-inflammatory cytokines and chemokines, among which are TNF-α, IL-12, IL-6 and CCL2 [7]. At the other extreme, “M2 microglia” refer to the states of alternative activation and these cells promote anti-inflammation and tissue repair [8]. Research of M1/M2 paradigm of microglial activation gains great attention. In this study, we focus on the roles of classical microglial activation in an attempt to disclose the mechanisms of neuroinflammation.

One of the potential mechanisms of inflammatory responses requires the involvement of CD200 (OX-2), a type I membrane glycoprotein, which is widely distributed and expressed on neurons, endothelium, and lymphocytes [9]. The corresponding receptor, CD200R (OX-2R), belonging to the immunoglobulin superfamily, is primarily expressed by myeloid cells and a subset of T and B cells [10]. Normal CD200-CD200R signaling maintains microglia in a quiescent state. Publications show that disruption of CD200-CD200R interaction primes microglia to abnormal activation and consequent pathological changes, such as experimental autoimmune encephalomyelitis (EAE), experimental autoimmune uveoretinitis (EAU) and Parkinson’s disease [11–13]. A family of the mouse CD200Rs (murine CD200R1, R2, R3, and R4) exhibit potential N-glycosylated sites, among which CD200R1 is highly glycosylated, containing ten potential N-glycosylation sites [14]. However, the functional role of N-glycosylation of CD200R1 remains to determine.

As a post-translational modification, N-glycosylation of proteins usually occurs at the sequence Asn-Xaa-Ser/Thr, where Xaa is any amino acid residue except proline [15, 16]. It is the consensus of academic circles that the glycosylation of proteins act a pivotal part in maintaining the organism homeostasis [17]. In the CNS, glycosylation of proteins is involved in a wide variety of biology functions including neuronal growth and survival [18]. N-linked glycosylation has been suggested to play an essential role in the process of microglia activation in CNS inflammation [19]. Several N-acetylglucosamine (NAG) moieties was observed at residues Asn44, Asn93 and Asn192 (N44, N93 and N192) of CD200R1. It is important to note that NAG observed by Hatherley et al. [20] was counted from extracellular regions of CD200R1, which reminded us that N20 referred to N44 here. At the microcosmic level, NAG moieties were distant from the interface with the exception of N44 of the extracellular regions of CD200R1. Structurally, N44 is involved in the interaction [20]. Mutation of CD200R residues at the CD200-binding interface effectively inhibited ligand binding [21]. We chose to perform Asparagine to Glutamine substitution (N → Q) to study the functional role of N-glycosylation on CD200-CD200R engagement.

In the present study, we focused on the N-glycosylation modification of CD200R1 in microglia, and investigated the significance of N44 of extracellular regions of CD200R1 in mediating classical microglial activation. Our data showed that mutation of N44 of CD200R1 disrupted CD200-CD200R1 interaction and facilitated the classical microglial activation, characterized by increased expression of M1 phenotype markers and release of pro-inflammatory cytokines.

## Materials and methods

### Preparation of cells

Cortical neuronal cultures were prepared from embryos of C57BL/6 mice (purchased from Nantong University). Experimental protocols and surgical procedures were carried out according to previously researches [22]. Briefly, the cerebral cortices were dissected and dissociated in PBS containing trypsin (0.25%) after removal of meningeal tissues. The reaction was stopped after 10 min. Primary cortical neurons were maintained in DMEM (Invitrogen, Dun Laoghaire, Ireland), in a humidified atmosphere containing 5% CO^2^/95% air at 37 °C. The medium was changed every 3 days.

Murine microglia cell line BV2 cells were cultured in DMEM (Dulbecco’s modified Eagle’s medium) containing 10% heat-inactivated fetal bovine serum (FBS, Hyclone, Logan, UT, USA). Cells were maintained at 37 °C in a 5% CO^2^ humidified atmosphere with a density of 2.4 × 10^6^ cells/cm^2^ for protein and RNA extraction. BV2 cells were treated with 100 ng/mL LPS (Sigma-Aldrich) for 24 h to generate activated cells.

In one series of experiments, BV2 cells were co-cultured with neurons; primary neuronal cells were added in suspension in DMEM in a ratio of 1:8 (neurons:glia). After treated with LPS (100 ng/mL) for 24 h, supernatant was collected and stored at − 80 °C for later analysis. To assessed the function of CD200-CD200R1 interaction, we blocked the interaction using an anti-CD200 blocking antibody (OX-90) (1 μg/mL; Serotec, UK) in the absence or presence of LPS in another series of experiments. In all cases, supernatant was collected to detect cytokines or LDH release and cells were harvested for later analysis.

### RNA isolation and quantitative RT-PCR

Total RNA was isolated from cells in the cultures using TRIZOL reagent. The cDNA synthesis was carried out with Revert AidTM RT Kit in a 25 μL reaction volumes following the protocol supplied by the manufacturer. Results were analyzed with the comparative CT method. Quantitative real time RT-PCR gene expression was generally normalized relative to the endogenous control genes (GAPDH) to give a relative quantification (RQ) value (2^-ΔΔCT^, where CT is the threshold number of target molecules). The following primers were used: iNOSfor: 5′- CCCTTCAATGGTTGG-TACATGG-3′; iNOSrev: 5′-ACATTGATCTCCGTGACAGCC-3′; CD86for: 5′- TTGTGTGTGTTCTGGAAACGGAG-3′; CD86rev: 5′-AACTTAGAGGCTGTT-GCTGGG-3′; Arg1for: 5′-TCATGGAAGTGAACCCAACTCTTG-3′; GAPDHfor: 5′-TGATGACATCAAGAAGGTGGTGAAG-3′; GAPDHrev: 5′-TCCTTGGAGGCCATGTGGGCCAT-3′; IL-1βfor: 5′-GAGCACCTTCTTTTCCTTCATCTT-3′; IL-1βrev: 5′-TCACACACCAGCAGGTTATCATC-3′; TNF-αfor: 5′-ATGGCCTCCCTCTCAGTTC-3′; TNF-αrev: 5′- TTGGTGGTTTGCTACGACGTG-3′. Samples were run for 50 cycles (95 °C for 15 s, 60 °C for 30 s, and 72 °C for 15 s).

### Inhibition of glycosylation and deglycosylation

Cultured cells were performed with 1 μg/mL Tunicamycin (TM, an inhibitor of N-linked glycosylation) in supernatant to completely block N-glycosylation. Moreover, in vitro enzyme inhibition experiments were performed. Protein lysate (50 μg of total protein) was incubated with 50 units of Endo H or 50 units of PNGaseF (New England Biolabs, USA) at 37 °C for 4 h following the protocol supplied by the manufacturer.

### Site-directed mutagenesis and plasmid DNA transfection

CD200R1 mutagenesis was performed using a primer pair: 5′- AGACACTGTAGTCTGCACTTGTGTCAGA -3′, 5′-TCTGACACAAGTGCAGACTACAGTGTCT -3′ at Asn 44 with substitution of Asparagine to Glutamine (N → Q). The complementary DNAs (cDNAs) encoding mouse CD200R1 and its mutants were cloned into the p3XFLAG-CMV-13 vector containing a FLAG tag. Mutants were amplified with Long-Taq-DNA polymerase (BIOSCI) from plasmids coding mouse FLAG-CD200R1. DNA plasmids were transfected into BV2 cells using Lipofectamine 2000 (Invitrogen) following the protocol supplied by the manufacturer.

### Immunoprecipitation (IP)

Cultured cells in the co-cultures were scraped into ice-cold PBS, and centrifuged at 4 °C for 10 min at 12,000 rpm. The pellet was re-suspended in 0.75 mL of lysis buffer and rotated at 4 °C for 2 h to insure lysis. Lysates were centrifuged at 14,000 rpm, 4 °C for 20 min. Supernatants were collected and pre-cleaned with 20 μL EZview Red Protein affinity gel (Sigma Chemical Co.) and 0.5 μg normal IgG for 2 h. Separately, 40 μL of the gel was preloaded with specific antibody for 2 h. Preloaded gel was washed once with lysis buffer, and added to precleared lysates and rocked overnight at 4 °C. The immunocomplexes were washed three times with lysis buffer and re-suspended in 15–20 mL 1 × Laemmli buffer (BioRad). After boiled, samples were electrophoresed onto the SDS-PAGE.

### Western blotting

We use western blotting to evaluate the inhibition of glycosylation and deglycosylation. After the indicated treatments as described above, cells were harvested, which were washed with PBS and lysed in a cell lysis buffer. The lysates were then centrifuged at 12,000 rpm, 4 °C for 15 min. After measuring the protein concentration of the supernatant, proteins were subjected to SDS-PAGE separation and electro-transferred to polyvinylidene difluoride membranes (PVDF, Millipore, Bedford, MA, USA). The membrane was then blocked in 5% nonfat milk diluted in 0.1% Tween 20-Tris-buffered saline (TBST; pH 7.4) for 2 h at room temperature. The membranes were incubated with primary antibodies at 4 °C overnight and then wash in TBST three times every 10 min. Followed by incubation with secondary antibody (1:2000; Southern Biotech) for 2 h at room temperature, membranes were washed again and revealed by an enhanced chemiluminescence system (ECL; Pierce Company, Woburn, MA, USA). Visualized profiles of bands were performed by ImageJ (NIH) to analyze the expression of proteins.

### Evaluation of cytokines by ELISA

The concentrations of inflammatory cytokines IL-1β and TNF-α were evaluated in samples of supernatant collected from cultured cells with different treatment described above using ELISA kit (Beyotime Biotechnology, CN). The assays were performed following the protocol supplied by the manufacturer. Cytokine concentrations were calculated by interpolation from the appropriate standard curve plotted.

### CD200 binding analysis

To assess the binding, we used biotinylated CD200 (R&D Systems) following the protocol supplied by the manufacturer. Levels of biotinylated CD200-bound CD200R1 could be obtained. Briefly, after transfected with CD200R1 WT or N44Q mutant, BV2 cells were incubated with various amounts of biotinylated CD200 for 1 h at 4 °C and then incubated with avidin-FITC reagent for 30 min at 4 °C in the dark. Cells were washed twice with PBS, treated with RDF buffer to remove unreacted avidin-fluorescein and resuspended for final flow cytometric analysis. Each sample was normalized by its individual negative staining control. Surface expression of the CD200R1 was determined by the analysis using monoclonal anti-FLAG antibody.

### Statistical analysis

All data were analyzed with Stata 7.0 statistical software. All values were expressed as mean ± SEM. One-way analysis of variance (ANOVA) followed by the Tukey’s post-hoc multiple comparison tests were used for statistical analysis. Each experiment consisted of at least three replicates per condition. Statistical significance was acceptable at the level of *p* < 0.05.

## Results

### N-glycosylation of CD200R1 and construction of CD200R1 mutant by the mutagenesis of potential N-glycosylation sites

**Fig. 1:**
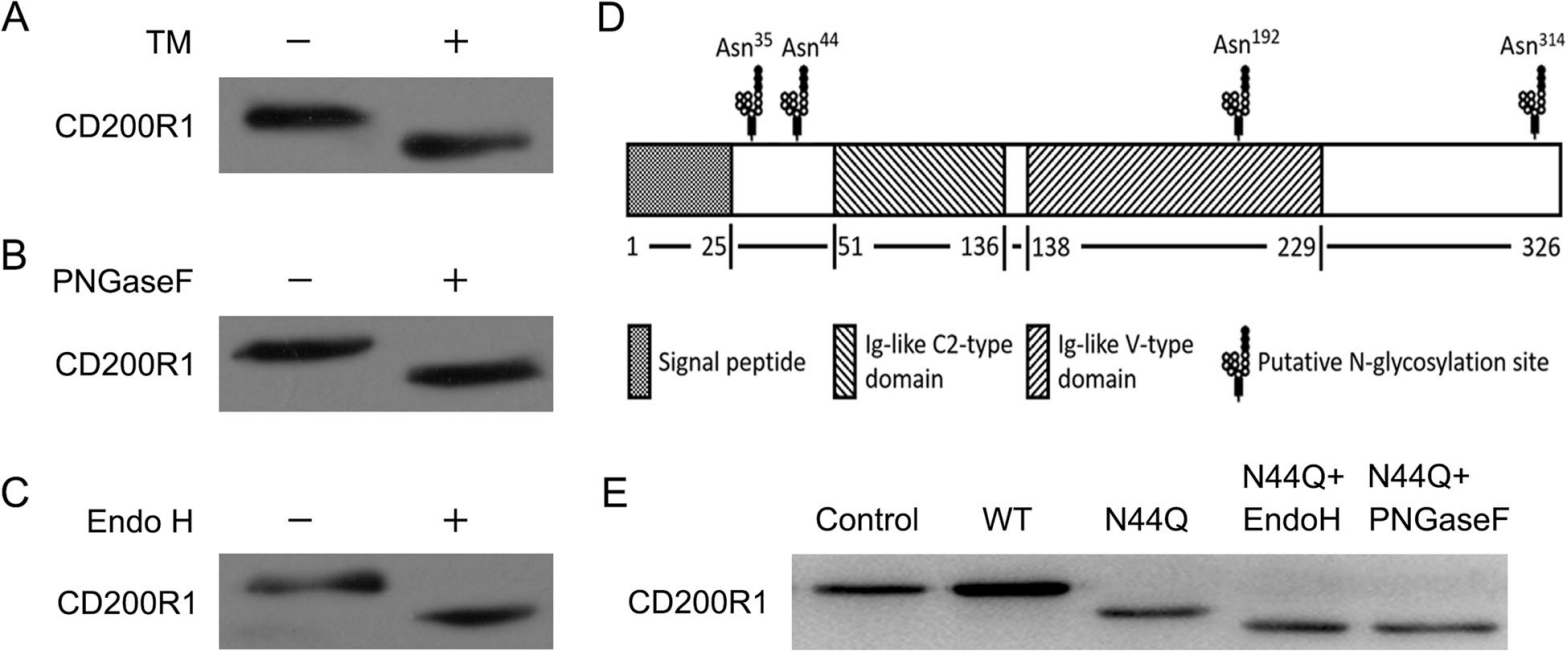
N-glycosylation of CD200R1 and construction of CD200R1 mutant. **a** The CD200R1 expressed in BV2 cells in the presence of N-glycosylation inhibitors (1 μg/mL TM) for 24 h was determined by western blotting analysis. **b** Cell lysates of BV2 cells were denatured and treated with PNGaseF and subjected to western blotting analysis. **c** Cell lysates of BV2 cells were denatured and treated with endoglycosidase (Endo H) and subjected to western blotting analysis. **d** Four potential N-glycosylation sites at Asn35, Asn44, Asn192, Asn314 were presented. We mutated the asparagine residue (N44) to glutamine (Q) to verify the N-glycosylation site on CD200R1. **e** N44Q mutant was generated and transiently expressed in BV2 cells. Additionally, some of cell lysates were treated by enzymatic digestion and then subjected to western blotting analysis. Error bars indicate ± SEM

To analyze the N-glycosylation of CD200R1, we abolished the addition of N-linked glycans with Tunicamycin (TM). TM treatment induced a mobility shift of the protein band revealed by western blotting analysis of BV2 cells extracts (Fig. 1a). N-linked carbohydrate moieties were enzymatically removed from CD200R1 by peptide N-glycosidase F (PNGaseF) treatment, which cleaves all forms of N-linked oligosaccharides, or endoglycosidase H (Endo H) treatment, which cleaves high-mannose and hybrid oligosaccharides (Fig. 1b and c). Bioinformatic prediction was employed to examine the N-glycosylation sites on CD200R1. Potential N-glycosylation profiles of CD200R1 were presented. There are four sites at Asn35, Asn44, Asn192 and Asn314 (Asn-X-Ser/Thr) as predicted by NetNGlyc 1.0 (Fig. 1d). Recent research has claimed three observed NAG moieties, among which N44 lies at the periphery of the interaction interface [20]. Here we mutated the asparagine residue (N44) to glutamine (Q) to verify the N-glycosylation site on CD200R1. N44Q mutant was generated and expressed in BV2 cells. As monitored by western blotting analysis, glycomutant decreased the molecular mass of CD200R1 band (Fig. 1e). These data suggested that CD200R1 was N-glycosylated on Asn44. Additionally, N44Q transfection showed the remain of glycosylation. Enzymatic digestion of N44Q transfection presented a minor change of molecular mass (Fig. 1e).

### Classical microglial activation in BV2 cells

**Fig. 2:**
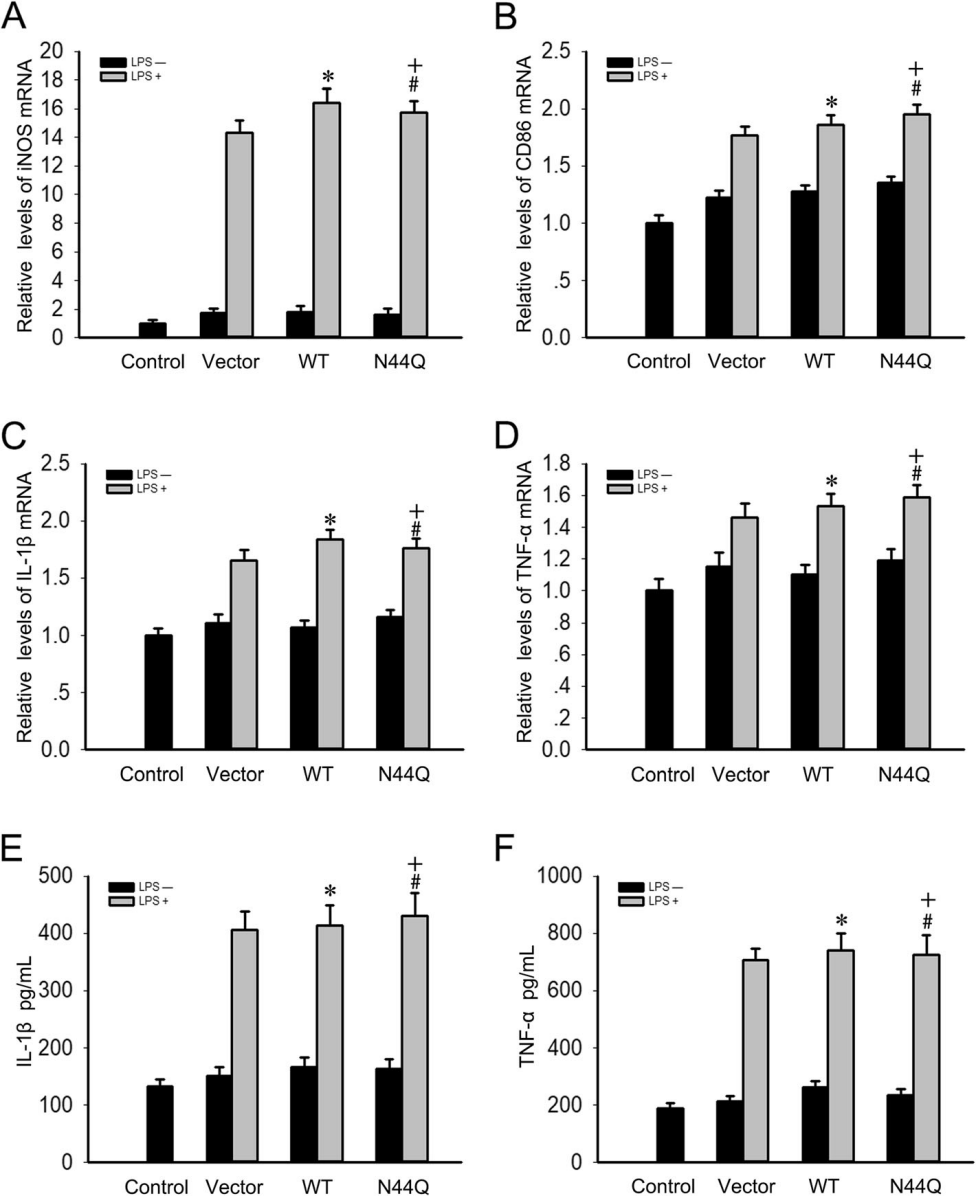
Classical microglial activation in BV2 cells. BV2 cells were treated with LPS (100 ng/mL) or PBS for 24 h to induce M1 phenotype. BV2 cells were transiently transfected with WT or N44Q mutant of CD200R1 to illustrate the effect of mutagenesis. **a**, **b** LPS induced iNOS mRNA and CD86 mRNA and the changes between WT and N44Q mutant did not reach statistically significant difference (^*^p < 0.01; ^*+*^*p* < 0.01; ^*#*^*p* > 0.05, N44Q vs WT; ANOVA; *n* = 3). **c**, **d** IL-1β mRNA and TNF-α mRNA were upregulated when BV2 cells were treated with LPS (^*^p < 0.01; ^*+*^*p* < 0.05; ^*#*^*p* > 0.05, N44Q vs WT; ANOVA; *n* = 3). **e**, **f** Similar results were obtained by ELISA (^*^p < 0.01; ^*+*^*p* < 0.05; ^*#*^*p* > 0.05, N44Q vs WT; ANOVA; *n* = 3). Error bars indicate ± SEM

In an attempt to analyze classical microglial activation, BV2 cells were exposed to LPS (100 ng/mL) to induce M1 phenotype. The data presented here showed that iNOS mRNA and CD86 mRNA increased in M1 cells (in response to LPS) (Fig. 2a and b). To estimate the effect of mutagenesis, BV2 cells were transiently transfected with WT or N44Q mutant of CD200R1. No statistically significant difference was found in mRNA levels of WT and N44Q mutant (*P* > 0.05) (Fig. 2a and b). We then examined the release of pro-inflammatory cytokines. Levels of IL-1β mRNA and TNF-α mRNA were upregulated in response to LPS (Fig. 2c and d). As expected, no statistical difference was found between WT and N44Q by ELISA (Fig. 2e and f). These data indicated that BV2 cells transfected with N44Q mutant alone showed no inhibitory effects.

### Neurons attenuate LPS-induced classical microglial activation

**Fig. 3:**
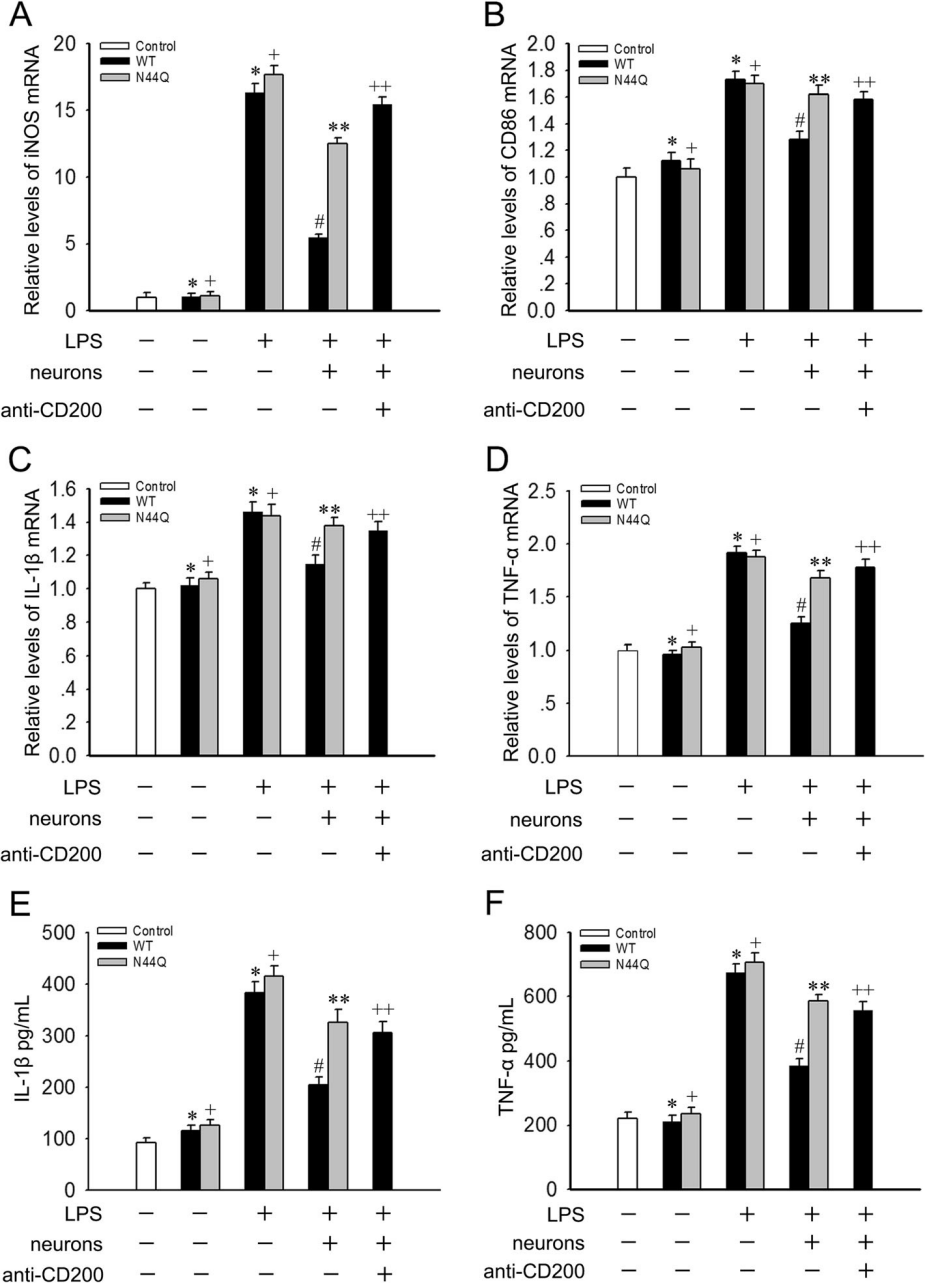
N44Q mutant involved in CD200-CD200R1 interaction and LPS-induced classical microglial activation. We established neuronal-microglia co-cultures to explore the effect of N44Q mutant. **a**, **b** LPS significantly increased iNOS mRNA and CD86 mRNA in BV2 cells. The addition of neurons attenuated LPS-induced classical microglial activation. Notably, the present of anti-CD200 antibody blocked the modulating effect of neurons. BV2 cells transfected with N44Q mutant exerted similar effects like anti-CD200 antibody (^*^p < 0.01; ^*+*^*p* < 0.01; ^*#*^*p* < 0.01, WT + LPS + neurons vs WT + LPS; ^**^p < 0.01; N44Q + LPS + neurons vs N44Q + LPS; ^*++*^*p* < 0.01, N44Q + LPS + neurons + anti-CD200 antibody vs N44Q + LPS + neurons; ANOVA; *n* = 5). **c**, **d** IL-1β mRNA and TNF-α mRNA were examined. The LPS-induced effects were reversed in the presence of neurons. Importantly, the addition of anti-CD200 antibody abrogated the modulating effect of neurons, consistent with the cells transfected with N44Q mutant (^*^p < 0.01; ^*+*^*p* < 0.01; ^*#*^*p* < 0.05, WT + LPS + neurons vs WT + LPS; ^**^p < 0.01; N44Q + LPS + neurons vs N44Q + LPS; ^*++*^*p* < 0.01, N44Q + LPS + neurons + anti-CD200 antibody vs N44Q + LPS + neurons; ANOVA; *n* = 5). Similar results were obtained by ELISA (^*^p < 0.01; ^*+*^*p* < 0.01; ^*#*^*p* < 0.01, WT + LPS + neurons vs WT + LPS; ^**^p < 0.01; N44Q + LPS + neurons vs N44Q + LPS; ^*++*^*p* < 0.01, N44Q + LPS + neurons + anti-CD200 antibody vs N44Q + LPS + neurons; ANOVA; *n* = 5). Error bars indicate ± SEM

We next assessed the effect of LPS on BV2 cells with a neuronal-microglia co-culture system as previously described [23, 24]. To further explore the role of N44 in modulating microglial activation, BV2 cells were incubated in the presence or absence of LPS, and co-cultured with neurons (which expressed CD200). LPS stimulation significantly increased iNOS mRNA and CD86 mRNA and the effect was attenuated by addition of neurons (Fig. 3a and b). Interestingly, we found that the attenuating effect of neurons was partially inhibited by including anti-CD200 blocking antibody during incubation in a separate experiment. BV2 cells transfected with N44Q mutant gained similar results (Fig. 3a and b). Levels of iNOS mRNA and CD86 mRNA were analyzed (Fig. 3c and d). The expressions of IL-1β and TNF-α were sharply increased after LPS treatment. This effect was attenuated by co-culture with neurons, whereas further treatment with anti-CD200 blocking antibody reversed the downregulation of expressions of IL-1β and TNF-α by neurons (Fig. 3e and f). BV2 cells transfected with N44Q mutant abolished the neuroprotective effects. Thus, effects exerted by N44Q mutant and CD200-CD200R1 dysfunction were analogous. It reminded us that glycosylation at N44 may be associated with microglial activation through a certain way by CD200-CD200R1 interaction.

### Blockage of CD200-CD200R1 interaction induced neuronal injury

**Fig. 4:**
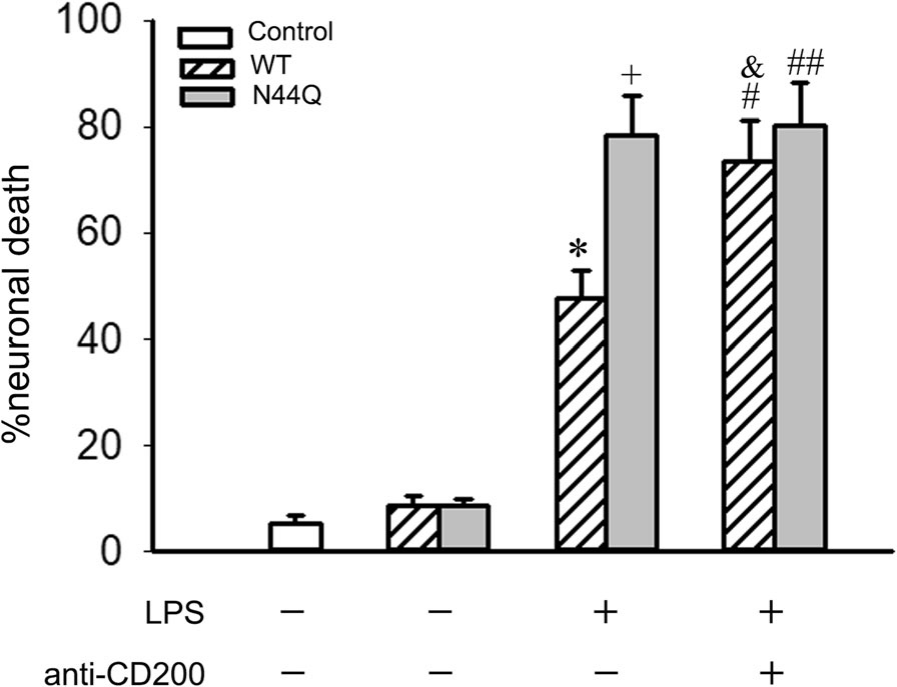
N44Q mutant enhanced neuronal death. N44Q mutant enhanced neuronal death: role of CD200-CD200R dysfunction. Resting or LPS stimulated BV2 cells transfected with WT or N44Q were cultured with neurons. Cortical neurons were co-cultured with BV2 cells for up to 24 h and neuronal death was estimated by LDH measurement in the media. The increased neuronal death induced by LPS stimulated BV2 cells was enhanced by the treatment with anti-CD200 antibody (^*^p < 0.01, WT + LPS vs WT; ^*+*^*p* < 0.01, N44Q + LPS vs N44Q; ANOVA; *n* = 3). BV2 cells transfected with N44Q mutant leaded to the reinforced neuronal death (^*^p < 0.01; ^*+*^*p* < 0.01; ANOVA; n = 3; ^*#*^*p* < 0.05, WT + LPS + anti-CD200 vs WT + LPS; ^*##*^*p* > 0.05, N44Q + LPS + anti-CD200 vs N44Q + LPS; ^&^*p* > 0.05, WT + LPS + anti-CD200 vs N44Q + LPS). Error bars indicate ± SEM. These data suggested N44Q mutant impaired CD200-CD200R1 interaction and subsequently leaded to microglial activation and neuronal death

To investigate the relevance of N-glycosylation in microglia-induced neurotoxicity, we analyzed the effect of CD200-CD200R1 interaction on neuronal death using a neuronal-microglia co-culture system. In all cases, neuronal death was quantitatively assessed by measurement of lactate dehydrogenase (LDH) release into the supernatant after 24 h of co-culture with microglial BV2 cells as previously reported [25]. As shown (Fig. 4), the increased neuronal death by LPS-stimulated microglia was enhanced by the treatment with anti-CD200 antibody. Interestingly, we found that BV2 cells transfected with N44Q mutant showed equivalent neuronal death comparable to addition of anti-CD200 antibody.

### Defective of N-glycosylation suppresses CD200-binding affinity

**Fig. 5:**
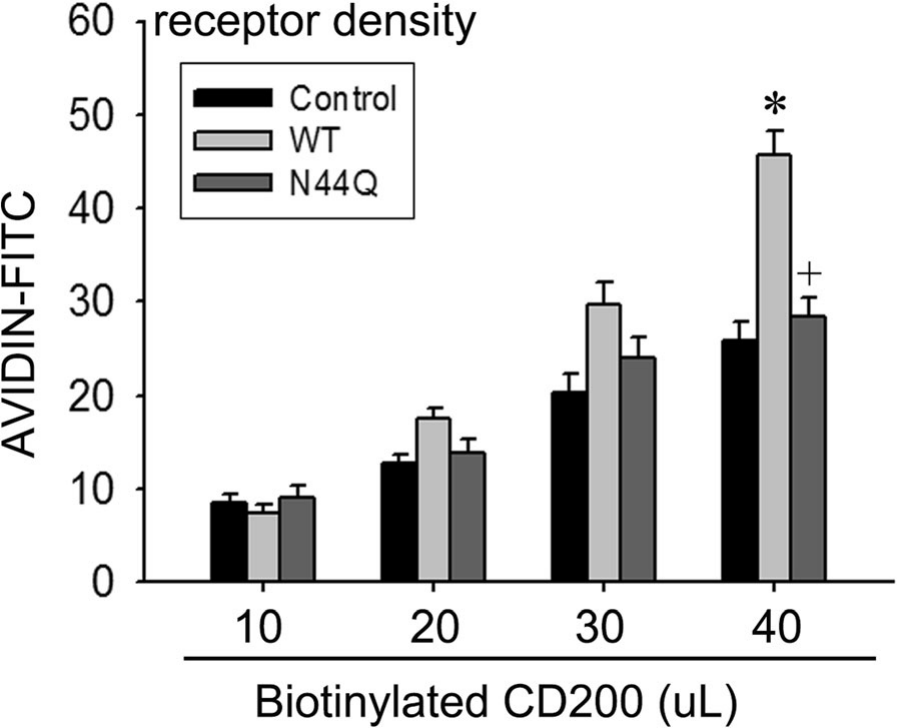
N44Q mutant impaired CD200-CD200R1 interaction. Representative flow cytometric analysis of receptor density on the cell surface. Various amounts of biotinylated CD200 (10–40 μL) were added to 10^5^ BV2 cells that were transiently transfected with empty vector, WT, or N44Q mutant. The numbers of biotinylated CD200R-bound CD200 were quantified using biotinylated CD200. Data are presented as means ± SEM (^*^ *p* < 0.05, WT vs control; ^*+*^*p* < 0.05, N44Q vs WT)

Previous studies have demonstrated that the N44 is located at the extracellular region of mouse CD200R1, which is essential for CD200-CD200R1 engagement (Hernangomez et al., 2012). Therefore, it is reasonable to presume that N44Q mutant may impair CD200-CD200R1 interaction, which subsequently led to neuronal injury. To testify this hypothesis, we performed a ligand binding assay to describe the binding affinity. BV2 cells expressing WT or N44Q mutant were treated with biotinylated CD200 and then examined by flow cytometric analysis (Fig. 5). Cells transfected with WT showed significant higher ligand binding affinity compared with mock. As expected, ligand binding in the N44Q mutant-transfected BV2 cells was comparable with that of the mock-transfected control cells. Ligand binding affinity was assessed by immunoprecipitation (IP) at a molecular level (Additional file 1: Figure S1). Cells were harvested 24 h after the co-cultures system established. Co-cultures were separated into two groups: a group of BV2 cells with WT and group of BV2 cells with N44Q. Western blotting analysis suggested that CD200 bind more CD200R1 with WT than CD200R1 with N44Q, which indicated the weak affinity binding to CD200R1 in the group of N44Q. These data suggested that N44Q mutant suppressed CD200-CD200R1 interaction.

## Discussion

In CNS inflammation, microglia become rapidly activated in response to injury while regulatory immune inhibitory molecules contribute to avoid its detrimental effects [25]. CD200R1 is a membrane glycoprotein, mostly expressed constitutively on cells of the myeloid lineage including microglia [26]. The corresponding ligand, CD200, is widely distributed on neurons, endothelium, and lymphocytes in the adult CNS [9]. The structure of CD200R and its implications for topology have already been explored thoroughly [20, 27]. However, the underlying biological functions of the glycosylation of CD200R1 are remain to be fully delineated. CD200R1 is N-linked glycosylated at N44 of extracellular regions and this site was important for ligand binding. An inhibitory effect exerted by normal CD200-CD200R signaling was abolished with N44Q mutant of CD200R1. Our data contained here can be summarized as following: N-glycosylation of CD200R1 was involved in its ligand binding and maintenance of microglia in a quiescent state. Our findings suggest a new model for regulation of CNS inflammation and offer directions where should we focus our efforts in the future.

It is generally accepted that glycosylation plays a pivotal role in the function of a glycoprotein [15]. N-glycosylation is an essential posttranslational modification during the maturation and secretion processes of many proteins, which exerts effects on cell-cell recognition, signaling and other cellular processes [19, 28, 29]. In the present study, we focused on CD200R1, investigating the role of N-glycosylation in ligand-receptor binding. Herein, we blocked the addition of N-linked glycans with TM, obtained by western blotting analysis (Fig. 2). Wright et al. reported that N-linked carbohydrate moieties were enzymatically removed from purified rat CD200R by PNGaseF treatment [30], and our results showed that the N-glycans of mouse CD200R1 were PNGaseF and Endo H sensitive, indicating that CD200R1 N-glycosylation sites were occupied with high-mannose or complex-type glycans. Next step of the research was to analyze the role of the N-glycosylated sites.

As an immune inhibitory molecule, CD200R1 binds to CD200, ultimately leading to inhibition of classical activation of macrophages and microglia [23]. Microglia respond to different stimuli by adopting different phenotypes indicative of their broad array of functions and morphology [31]. Microglial activation in the CNS is heterogeneous, which can be described as a functional dichotomy, termed classical (M1) and alternative (M2) activation [5]. One of the endogenous mechanisms of immune regulation requires involvement of the ligand-receptor interaction. CD200-CD200R is a ligand-receptor pair controlling microglial activation, providing a cell-cell contact negative regulatory signal for microglia [12, 22]. To evaluate the effects of the CD200R1 on murine microglial activation, we established neuronal-microglia co-cultures to explore the involvement of CD200-CD200R1 interaction. Here, we pushed microglia to classical activation with the treatment of LPS and examined the released cytokines of M1 cells. Markers of M1 cells and pro-inflammatory cytokines were highly induced. As expected, CD200R1 engagement, in the presence of neurons providing CD200, decreased the production of the M1 markers and pro-inflammatory cytokines. Notably, addition of anti-CD200 antibody abrogated the modulating effect of neurons. Publications have illustrated that deficits in the CD200-CD200R1 interaction exacerbate microglial activation [12, 32]. To analyze the role of the N-glycosylation of CD200R1, asparagine (N) residue at Asn44 was replaced with glutamine (Q) residue using site-directed mutagenesis. Our data showed that the site of consensus Asn-X-Ser/Thr sites of CD200R1 was glycosylated (N44).

**Fig. 6:**
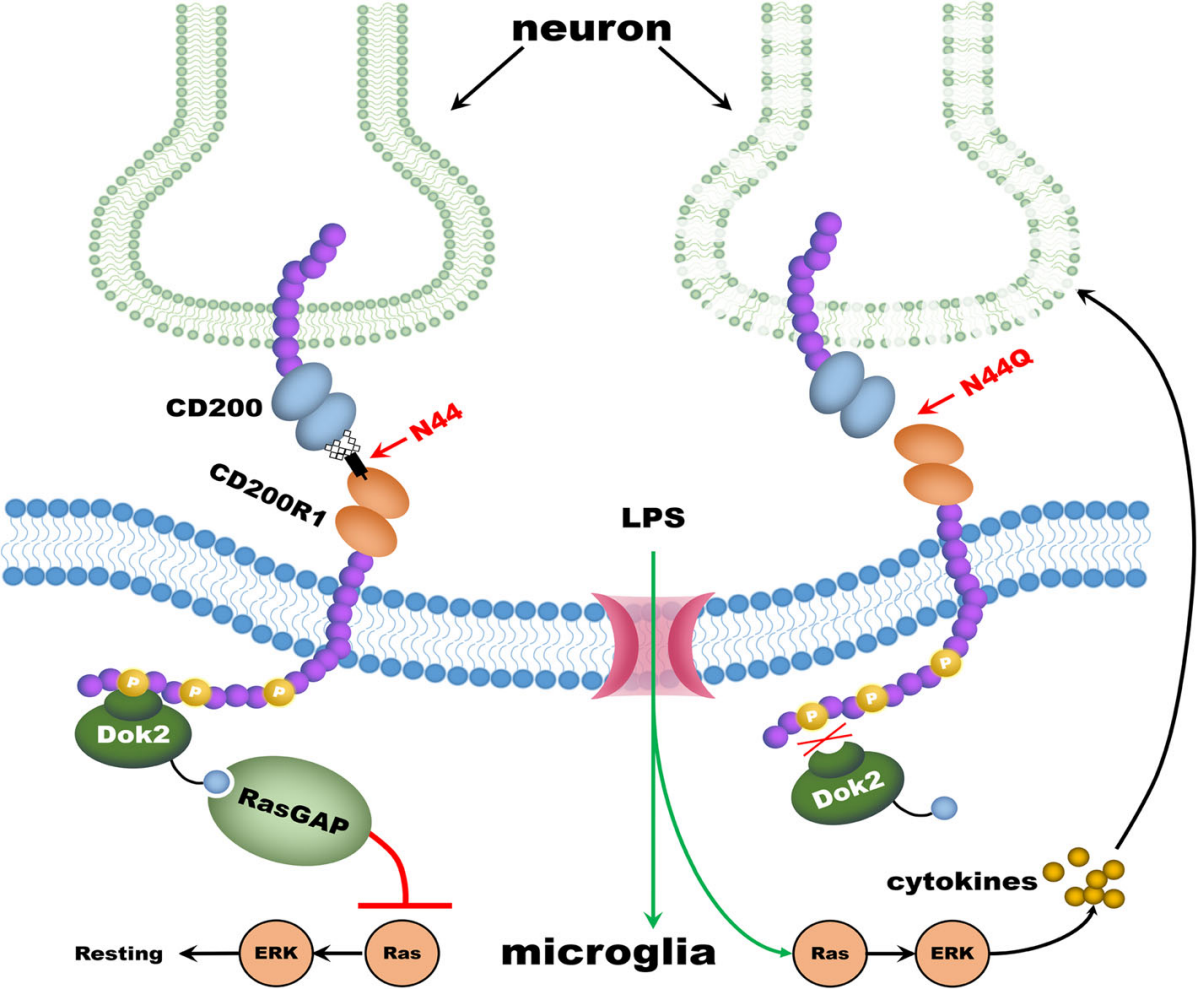
Presumptive mechanism of CD200–CD200R1 interaction between neurons and microglia. CD200 bind CD200R1 through N-terminal amino acid, leading to the activation of CD200R. The tyrosine residues of CD200R recruits DOK2 and RasGAP, ultimately resulting in the inhibition of Ras activation and reduce of multiple anti-inflammatory signals. Blocked interaction by N44Q mutant inhibits the signaling of CD200R inactivates downstream proteins, leading to the increase of multiple pro-inflammatory signals under LPS stimulation. P: Phosphotyrosine

Note that LPS has no direct effect on neuronal cell death. However, some microglial cell activators can be directly neurotoxic [33]. When cells undergo death, definable patterns of cell death are produced. Necrotic cell death or necrosis is an accidental type of cell death and always caused by pathological factors. In contrast to necrosis, Apoptotic cell death or apoptosis is an active cellular process and can occur naturally during normal brain development. Necrosis usually initiates an inflammatory response whereas apoptosis not [34–36]. Apoptosis is one of the key regulatory mechanisms in tissue modeling and the establishment of neuronal connections. In the study, neuron death was secondary to BV2 cells activation. LPS-stimulated BV2 cells expressed inflammatory cytokines and had effects over neuron death. CD200R has a cytoplasmic tail which contains three tyrosine residues, one of which forms part of a NPXY motif. Upon binding to its ligand, the tyrosine residues of CD200R on resting microglial cells become phosphorylated and adaptor proteins down-stream of tyrosine kinase (DOK2) is recruited. This ultimately leads to activation of RasGAP. This process inhibits Ras activation, resulting in inhibition of other downstream inflammatory signals (Fig. 6). When BV2 cells were transfected with N44Q mutant, CD200-CD200R interaction was disrupted, which leaded to inhibit of the signaling of CD200R1 and blockage of subsequent cascade stage. We analyzed the effect of N44Q mutant on neuronal death, which was estimated by LDH measurement in the media. Addition of neurons elicited a significant decrease in the level of neuronal death that could be completely abrogated when microglia were treated with anti-CD200 antibody or expressed N44Q mutant. Glycan at N44 was a bridge of CD200-CD200R1 engagement, which suggested that the ligand binding regulated microglial activation in a different way.

As publications showed, the failure of the activating receptor to bind CD200 resides in subtle changes around the interface [20, 21]. Our results showed that elimination of N-glycosylation at Asn44 suppressed ligand-binding affinity. Thus, we reported that N-glycosylation of CD200R1 enhanced its binding to CD200, holding microglia in a quiescent state.

In summary, we set out to study the functional role of N-glycosylation of CD200R1 in classical microglial activation and demonstrate that N44Q mutant of CD200R1 inhibited the CD200-CD200R1 interaction, subsequently triggering classical microglial activation. Our data indicates that N44 is an important binding site for CD200R1, which is essential for inhibitory CD200R1 signaling and may contribute to the maintenance of microglia in a quiescent state. Manipulating the N-glycosylation of CD200R1 could provide a novel therapeutic target for treating CNS inflammation.

## Conclusion

To conclusion, our current study demonstrates that CD200R1 is N-glycosylated at N44. Mutation of N44Q could disrupt CD200-CD200R1 interaction and exert impact on microglial activation. Our study therefore supports an available application of treating CNS inflammation.

## Additional file

Additional file 1: **Figure S1.** CD200R1 interacted with CD200. Cells were harvested as described in “Materials and Methods” and immunoprecipitation (IP) was performed to detect CD200-CD200R1 interaction at a molecular level. BV2 cells were transfected with FLAG-WT or FLAG-N44Q and co-cultured with neurons. Proteins in the cell lysates and immunoprecipitated proteins were subsequently analyzed by western blotting analysis. Data showed that CD200 bind more CD200R1 with WT than CD200R1 with N44Q. (TIF 279 kb)

## Abbreviations

Asn: Asparagine
CD200R1: CD200 receptor 1
CNS: central nervous system
EAE: experimental autoimmune encephalomyelitis
EAU: experimental autoimmune uveoretinitis
Endo H: endoglycosidase H
IFN-γ: interferon-γ
LPS: Lipopolysaccharide
NAG: N-acetylglucosamine
PNGaseF: peptide N-glycosidase F
TM: Tunicamycin
