# Supplementary figures and images for: The role of N-glycosylation of CD200-CD200R1 interaction in classical microglial activation
Source: J Inflamm (Lond). 2018 Dec 19;15:28. doi: 10.1186/s12950-018-0205-8 (PMC6300008; doi:10.1186/s12950-018-0205-8)

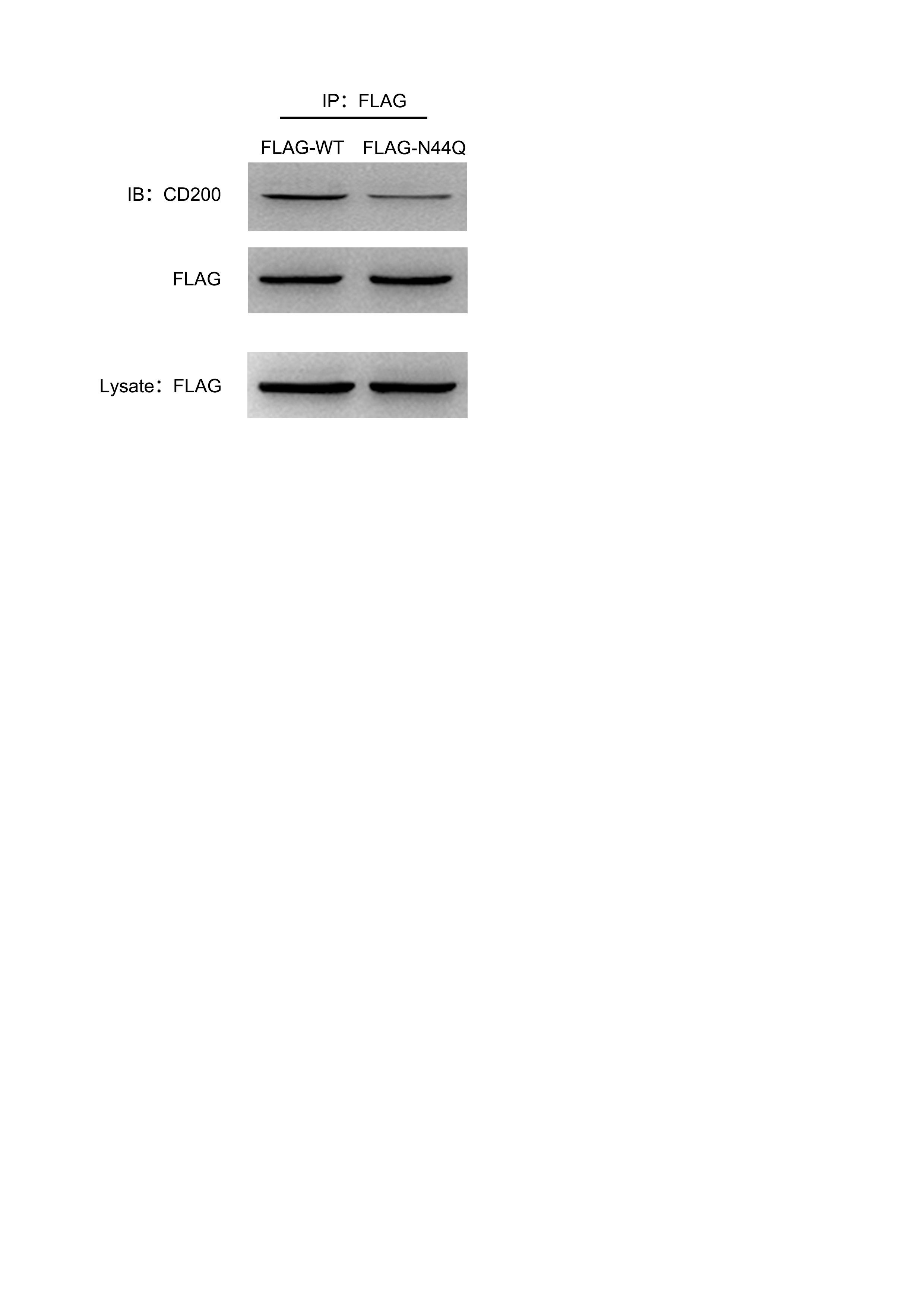

Supplement: Supplementary file 1 — Figure S1. CD200R1 interacted with CD200. Cells were harvested as described in “Materials and Methods” and immunoprecipitation (IP) was performed to detect CD200-CD200R1 interaction at a molecular level. BV2 cells were transfected with FLAG-WT or FLAG-N44Q and co-cultured with neurons. Proteins in the cell lysates and immunoprecipitated proteins were subsequently analyzed by western blotting analysis. Data showed that CD200 bind more CD200R1 with WT than CD200R1 with N44Q. (TIF 279 kb) [file 12950_2018_205_MOESM1_ESM.tif]
